# PEG-mediated transduction of rAAV as a platform for spatially confined and efficient gene delivery

**DOI:** 10.1186/s40824-022-00322-1

**Published:** 2022-12-02

**Authors:** Liang Zou, Jinfen Wang, Ying Fang, Huihui Tian

**Affiliations:** 1 CAS Center for Excellence in Nanoscience, National Center for Nanoscience and Technology, Beijing, 100190 China; 2 CAS Center for Excellence in Brain Science and Intelligence Technology, Institute of Neuroscience, Chinese Academy of Sciences, Shanghai, 200031 China; 3 University of Chinese Academy of Sciences, Beijing, 100049 China

**Keywords:** Adeno-associated virus, Polyethylene glycol, Transgene expression, Transduction, Neural circuit

## Abstract

**Background:**

Recombinant adeno-associated viruses (rAAV) are commonly used vectors for gene delivery in both basic neuroscience and clinical applications due to their nonpathogenic, minimally immunogenic, and sustained expression properties. However, several challenges remain for the wide-scale rAAV applications, including poor infection of many clinically important cell lines, insufficient expression at low titers, and diffusive transduction in vivo.

**Methods:**

In this work, PEG, which is a safe and non-toxic polymer of ethylene oxide monomer, was applied as an auxiliary transduction agent to improve the expression of rAAV. In detail, a small dose of PEG was added into the rAAV solution for the transgene expression in cell lines in vitro, and in the central nervous system (CNS) in vivo. The biocompatibility of PEG enhancer was assessed by characterizing the immune responses, cell morphology, cell tropism of rAAV, neuronal apoptosis, as well as motor function of animals.

**Results:**

The results show that small dose of PEG additive can effectively improve the gene expression characteristics of rAAV both in vitro and in vivo. Specifically, the PEG additive allows efficient transgene expression in cell lines that are difficult to be transfected with rAAV alone. In vivo studies show that the PEG additive can promote a spatially confined and efficient transgene expression of low-titer rAAV in the brain over long terms. In addition, no obvious side effects of PEG were observed on CNS in the biocompatibility studies.

**Conclusions:**

This spatially confined and efficient transduction method can facilitate the applications of rAAV in fundamental research, especially in the precise dissection of neural circuits, and also improve the capabilities of rAAV in the treatment of neurological diseases which originate from the disorders of small nuclei in the brain.

**Supplementary Information:**

The online version contains supplementary material available at 10.1186/s40824-022-00322-1.

## Introduction

Adeno-associated virus (AAV) is a nonpathogenic and replication-deficient parvovirus with a ca. 4.7-kilobase single-stranded DNA genome [1, 2]. Combined with molecular genetic techniques, recombinant AAV (rAAV) has proved to be a safe and minimally immunogenic means to achieve stable and sustained transgene expression [3–5] across a wide host range, which provides a highly promising vector system for both fundamental research and clinical applications. Recently, rAAV has been extensively used as vectors for gene transfer both in vitro and in vivo, especially in the central nervous system (CNS), e.g., cell-based gene therapy [6], gene modulating techniques [7], neural circuit analysis [8, 9], and disease model development [10, 11]. However, several challenges still remain in the wide-scale applications of rAAV. For example, rAAV shows poor infection efficiency in many clinically important cell lines [1, 12]. In addition, the in vivo applications of rAAV also face many challenges. Firstly, high-titer rAAV solutions, which are often needed to achieve efficient transgene expression in targeted brain regions, can cause neurotoxicity and immune responses [13–18]. Secondly, the rapid diffusion of viral solutions can result in large and coarse transduction volumes and unintended gene modulation of neighboring brain tissues [19]. The unintended transgene expression confounds the results of neural circuit dissection and also compromises the therapeutic effects of neurological diseases, e.g., Parkinson’s disease, that originate from the disorders of small nuclei in the brain [20–22]. Therefore, it is highly desirable to develop new strategies that can enable a spatially confined and efficient transgene expression of low-titer rAAV in vivo.

Previous efforts to improve the transduction efficacy of rAAV vectors have focused primarily on refining the vector system through capsid screening and genome modification [23, 24], which, however, is cumbersome and labor-intensive. In addition, during rAAV transduction, the synthesis of second-strand DNA has been considered to be a limiting step. Thus, self-complementary adeno-associated virus (scAAV) has been developed to overcome the limiting step in transduction [25]. Nevertheless, as the transgene cassette size of scAAV is limited to 2.3 kb, the practical applications of scAAV are limited. Therefore, novel and simple strategies that can effectively enhance the transgene expression of rAAV vectors are of great importance for their wide-scale applications. The development of auxiliary additives is another strategy to enhance rAAV transduction in the CNS. For example, chemical auxiliaries have been developed to enhance the transduction efficiency of rAAV in early studies through the inhibition of the activity of topoisomerase, the modulation of proteasome function, or the protection of the DNA structures [26, 27]. However, the cell toxicity and short enhancement validity of previous chemical auxiliaries have greatly limited their applications.

Polyethylene glycol (PEG) is a safe and non-toxic polymer of ethylene oxide monomers and has been approved for human use by U.S. food and drug administration (FDA) [28–30]. PEG or its derivatives have been extensively used as drug carrier, or covalently bonded to protein, peptides, enzymes, or small molecule agents for improving the drug delivery efficiency and therapeutic effects [31–35]. In addition, PEG has been a commonly used reagent in the precipitation and purification of lentivirus or adeno-associated virus [36]. In this work, PEG was applied as an auxiliary transduction agent to improve the expression of rAAV in vitro and in vivo. For in vitro transduction, the addition of PEG can significantly augment the infection efficiency of rAAV for all tested cell lines. For in vivo studies, the PEG additive can enable a spatially confined and efficient transgene expression of low-titer rAAV in the brain. This improvement could persist for months without measurable side effects, such as aggravated immune responses, abnormal cell morphology, alteration of rAAV tropism, cytotoxicity, and animal behavior alteration. The strategy of adding a minor dose of PEG as an auxiliary transduction agent can broaden the applications of rAAV in both scientific research and clinical therapy, especially when it is necessary to precisely and efficiently transfer genes to the small nuclei of brain.

## Methods

### Cell lines, animals and viruses

HeLa, HEK293T, K562, PC12, and C8-D1A cells were purchased from the Cell Resource Center of Chinese Academy of Medical Sciences. Male C57BL/6 mice were purchased from Vital River Laboratory Animal Technology Co., Ltd. The mice were individually housed in a temperature and humidity controlled environment under a 12 h:12 h light:dark cycle, with ad libitum access to food and water. All animal procedures were approved by the Animal Care and Use Committee of the National Center for Nanoscience and Technology, China. The mice were 7–8 weeks old and weighed 20–30 g at the time of viral injection. The rAAV vectors, including rAAV9-CMV::GFP, rAAV9-hSyn::EGFP, and rAAV9-GFAP::EGFP were purchased from Hanbio Co., Ltd.

### Chemical reagents and instruments

The phosphate-buffered saline was purchased from HyClone Laboratories (AE29246295) and the polyethylene glycol with molecular weight of 4000 was purchased from Sigma-Aldrich Co., LLC. (95904-250G-F). Dulbecco’s modified eagle medium, fetal bovine serum, penicillin, streptomycin and heat-inactivated horse serum were all purchased from Gibco Company. 4% paraformaldehyde was purchased from Biosharp Company (BL539A) and fluoromount G was purchased from Southern Biotechnology Associates, Inc. (A3420-MM70). Mouse primary antibody for staining NeuN and rabbit primary antibody for staining GFAP were purchased from Sigma-Aldrich Co., LLC. (MAB377, HPA056030). The goat anti-mouse IgG (H + L) secondary antibody 633 and the goat anti-rabbit IgG (H + L) secondary antibody 594 were purchased from ThermoFisher (A-21050 and A-11012). All reagents were of analytical grade and used without further purification. Cell Counting Kit-8 (CCK-8, CK04–10) was purchased from Dojindo, Japan.

Stereotaxic apparatus frame was purchased from RWD Life Science Co. Ltd. The rAAV solutions were injected through a 10-μL microinjector (1701RN, Hamilton) assembled with a G34 syringe needle (Hamilton) under the control of a PUMP 11 ELITE Nanomite (Harvard apparatus). Brian slices were sectioned from a freezing microtome (Leica Biosystem). The fluorescence images were acquired from a confocal microscope (Zeiss LSM710, Carl Zeiss). The movements of mice were recorded and analyzed with SuperMaze video tracking software purchased from Shanghai XinRun Information Technology Co., Ltd.

### In vitro transduction assays

HEK293T, HeLa, and C8-D1A cell lines were cultured in Dulbecco’s Modified Eagle Medium supplemented with 10% fetal bovine serum and 1% Penicillin/Streptomycin. K562 cell was cultured in RPMI-1640 supplemented with 10% fetal bovine serum and 1% Penicillin/Streptomycin. PC12 was cultured in RPMI-1640 supplemented with 5% fetal bovine serum, 10% heat-inactivated horse serum and 1% Penicillin/Streptomycin. The cell cultures were grown in a humidified incubator at 37 °C with 5% CO_2_. In our study, although different growth conditions were used for different cell types, the serotype and MOI of rAAV for each cell type were used under the same conditions, thus providing an internal control for comparative analysis.

In cell transduction experiments, rAAV9-CMV::GFP vectors were used for HEK293T, HeLa, K562, and PC12 cells, and rAAV9-GFAP::EGFP was used for C8-D1A cell. About 10,000 cells per well were plated in 1 mL of media in a 24-well plate (Cellvis, #P24–1.5H-N). Immediately after plating, the cells were infected with rAAV9-CMV::GFP/PEG solutions at the MOI of 5 × 10^5^ v·g/cell, and with PEG concentration of 3.6% (wt/vol). In the meanwhile, the cells of the control group were infected with rAAV9-CMV::GFP solutions alone at the MOI of 5 × 10^5^ v·g/cell. After 3-day infection, GFP expression was analyzed with confocal microscope using a 488-nm laser as the excitation source. To estimate the percentages of the infected cells, 20× magnification images were taken across multiple regions for HEK293T, HeLa, K562, and PC12 cells in random. The number of GFP-positive cells was counted with ImageJ software and three parallel tests were taken for each concentration of PEG. In each test, three random regions with over 500 cells for PC12, and over 1000 cells for HEK293T, HeLa, and K562 were counted for each well.

To investigate transduction efficiency of rAAV9-CMV::GFP on the dependence of PEG additive concentration, we infected HeLa, HEK293T, K562, and PC12 cells by rAAV9-CMV::GFP solutions accompanied with different amounts of PEG. The MOIs of the rAAV9-CMV::GFP in all culture wells were kept at 5 × 10^5^ v·g/cell, while the concentrations of the PEG were 0.2, 0.8, 1.6, and 3.6% (wt/vol), respectively. GFP expression was analyzed after 3-day infection as described above. For C8-D1A cell, the MOI of rAAV9-GFAP::EGFP was kept at 2 × 10^6^ v·g/cell, and the concentration of PEG additives were 1.6, 3.6, 5.4, and 7.2% (wt/vol), respectively.

### Surgery and stereotaxic injection

Mice were anesthetized with intraperitoneally administered pentobarbital sodium and fixed in a standard rodent stereotaxic apparatus frame with two ear bars. Chlortetracycline hydrochloride eye ointment was applied to both eyes to prevent eye drying. A heating pad was placed under the mouse to maintain its body temperature at 36–37 °C, and the anesthesia depth was monitored by testing the paw pinch reflex before the start of surgery. Two square holes of ca*.* 1 mm^2^ were drilled into the skull above the left and right M2 cortexes (anteroposterior, + 2.46 mm; mediolateral, ±0.84 mm; dorsoventral, − 1.50 mm) and the dura was removed carefully. rAAV and rAAV/PEG solutions were injected into the targeted brain region with an injection rate of 0.1 μL·min^− 1^, respectively. After injection, the syringe was left in place for 10 min before retracting the needle to avoid solution backflow. Then the holes on the skull were filled with silicon elastomer Kwik-Sil before sewing up the scalp. After surgery, the mice were allowed to recover on a 37 °C heating pad for 1 hour before returning to their home cages and an intraperitoneal injection of antibiotic drug was given to the mice for 3 days after the surgery.

### Tissue preparation, immunohistochemistry and imaging

At 3 or 8 weeks after rAAV injection and gene expression, the mice were anesthetized with an intraperitoneal injection of pentobarbital sodium, and transcardially perfused with 1× PBS and 4% paraformaldehyde (wt/vol), successively. After that, the mice were decapitated, and the brains were carefully removed. The brains were incubated in 4% paraformaldehyde overnight for post fixation and sectioned into 30-μm-thick slices using a freezing microtome. After that, the slices were mounted on glass slides, and fluoromount G was used to protect the fluorescent proteins from quenching. Last, confocal microscope images were acquired using a 488-nm laser as the excitation source.

For NeuN and GFAP staining, the brain slices were prepared following the same procedures described above. Then the slices were incubated in 0.3% Triton X-100 (wt/vol) at 25 °C for 15 min to increase the permeability of the membrane, and blocked with 3% bull serum albumin (wt/vol) for 1 h at room temperature to occupy the nonspecific binding sites. After that, the slices were transferred into primary antibodies (1:200 dilution for NeuN and 1:1000 dilution for GFAP) overnight at 4 °C. After being rinsed three times with 1× PBS, the slices were incubated in fluorophore-conjugated antibodies (1:1000 dilution) for 2 hours at room temperature. Then the slices were rinsed 3 times with 1× PBS and mounted on glass slides. As described above, fluoromount G was used to protect the fluorophores from quenching. Finally, the fluorescence images were acquired on a confocal microscope, and analyzed by ZEN and ImageJ software.

### Behavior assays

Eight wild-type mice were randomly divided into two groups. The control group of mice received an injection of untreated rAAV9-hSyn::EGFP solution, but the other group received an injection of 40% PEG-treated rAAV9-hSyn::EGFP solution in the M2 cortex (anteroposterior, + 2.46 mm; mediolateral, + 0.84 mm; dorsoventral, − 1.50 mm). At 3 weeks after injection and gene expression, the mice were placed in a standard open field behavioral chamber (50 × 50 cm^2^) and allowed to move freely for 15 minutes to adapt the experiment environment. After the acclimation, the movements of mice were recorded for 15 minutes at day and night individually with SuperMaze video tracking software. When replacing a mouse, spraying 75% alcohol to remove the smell inside the behavioral chamber was necessary. Finally, the average locomotion speeds and resting time were analyzed with SuperMaze video tracking software and used to assess the motor function of the mice.

## Results

### PEG-enhanced rAAV expression in vitro

We chose rAAV serotype 9 (rAAV9) to evaluate the effects of PEG additive on transgene expression because it has been widely used for gene delivery in the mammalian brain [37], especially well-suited for the transduction of neurons [38], and is currently being evaluated in clinical trials for the treatment of neurological disorders [39]. We examined the transduction efficiency of rAAV9 vectors encoding green fluorescent protein (GFP) under the control of the cytomegalovirus promoter (rAAV9-CMV::GFP) in four cell lines, including HeLa, HEK293T, K562 and PC12. HeLa is a type of cervical cancer cell, HEK293T is a human embryonic kidney cell, and K562 is a hematopoietic derived cell, all of which belong to human primary cells. Prior studies have shown that rAAV9 is moderately effective at transfecting HeLa and HEK293T cells but has no transduction in K562 cells [12]. In addition, we also examined the transduction efficiency of rAAV9 in PC12 cells because they constitute a widely used neuronal model system.

**Fig. 1:**
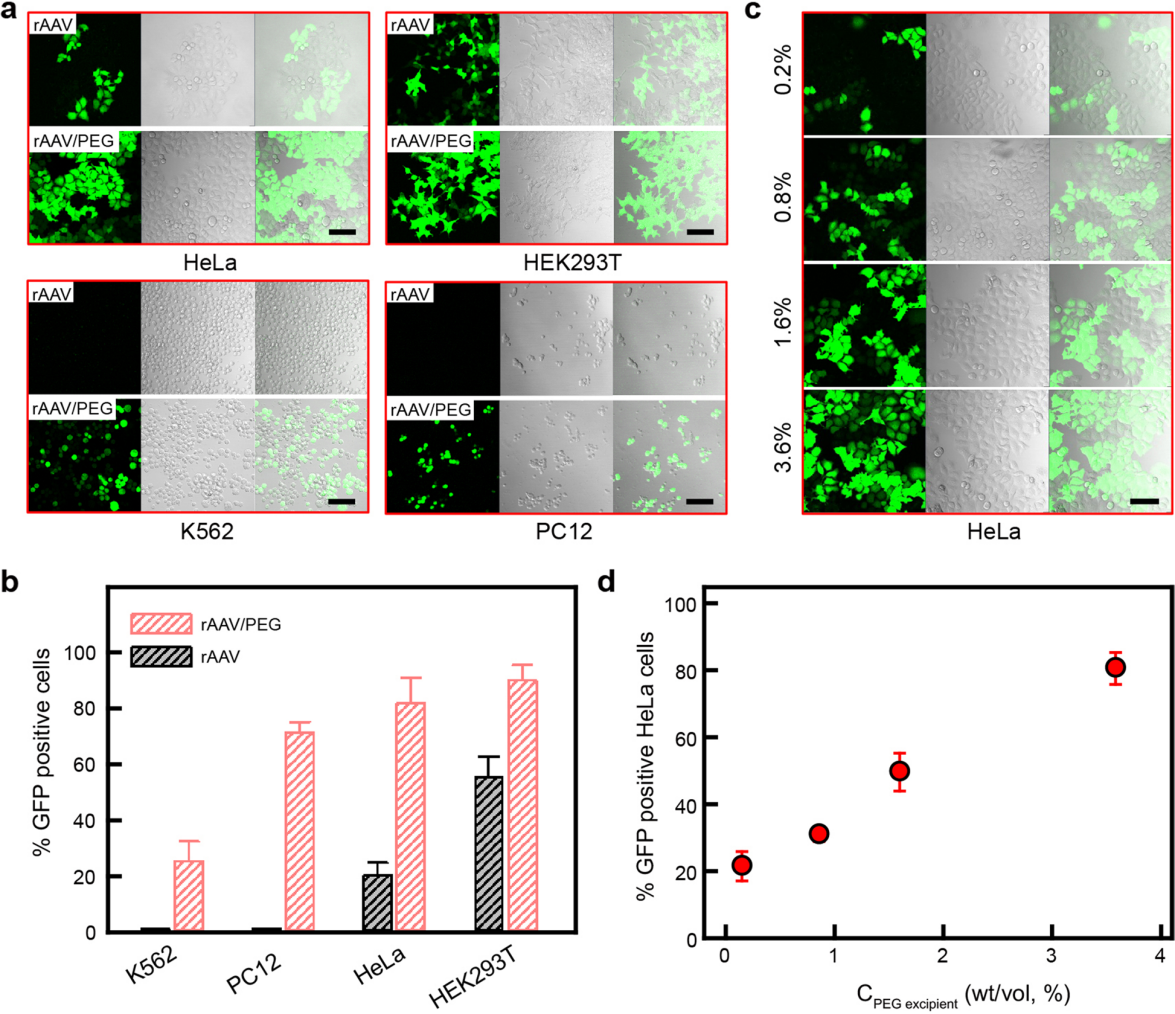
PEG-enhanced infection of rAAV-CMV::GFP vectors in vitro. **a** GFP expression in HeLa, HEK293T, K562 and PC12 cell lines transduced with untreated rAAV9-CMV::GFP (top) and rAAV9-CMV::GFP with 3.6% (wt/vol) PEG additive (bottom), respectively, at the MOI of 5 × 10^5^ v·g/cell. For each cell line, the left column shows the fluorescence images, the middle column shows the bright field images, and the right column shows the overlay images, respectively. Scale bars, 100 μm. **b** Quantitative analysis of the transduction efficiency of untreated rAAV9-CMV::GFP and rAAV9-CMV::GFP with 3.6% PEG additive, respectively, in different cell lines at the MOI of 5 × 10^5^ v·g/cell. *n* = 3 wells in each group. Data are represented as mean ± SD. **c** Transgene expression of rAAV9-CMV::GFP in HeLa cells with different concentrations of PEG additive. The MOI was kept at 5 × 10^5^ v·g/cell, and the concentrations of PEG additive were 0.2, 0.8, 1.6, and 3.6% (wt/vol) from top to bottom, respectively. Columns from left to right: green fluorescence, bright field, and overlay images. Scale bar, 100 μm. **d** Dependence of the transduction efficiency of rAAV9-CMV::GFP in HeLa cells on the concentration of PEG additive. The MOI was kept at 5 × 10^5^ v·g/cell. *n* = 3 wells in each group. Data are represented as mean ± SD

To evaluate the effects of PEG additive, rAAV9-CMV::GFP/PEG transduction solutions were added into cell cultures at an multiplicity of infection (MOI) of 5 × 10^5^ v·g /cell. Meanwhile, untreated rAAV9-CMV::GFP solutions were diluted to the same titer and added into cell cultures as a control. After a 3-day infection, the transduction efficiency was analyzed by measuring GFP fluorescence. As shown in Fig. 1a, in the presence of 3.6% (wt/vol) PEG additive, the rAAV9-CMV::GFP solutions induced greatly enhanced expression of GFP in HeLa and HEK293T cells in comparison with the control groups. More interestingly, GFP expression was detected in both K562 and PC12 cells incubated with rAAV9-CMV::GFP/PEG, whereas no GFP expression was observed in K562 or PC12 cells incubated with the rAAV9-CMV::GFP control solutions. We summarized the percentages of GFP-positive cells in Fig. 1b. When infected with untreated rAAV9-CMV::GFP solutions at an MOI of 5 × 10^5^ v·g/cell, the percentages of GFP-positive cells (defined as transduction efficiency) in HeLa and HEK293T cultures were 20 and 55%, respectively, similar to previous reports [12]. On the other hand, the transduction efficiency reached as high as 81 and 89%, respectively, for HeLa and HEK293T cells in the presence of 3.6% PEG additive under the same MOI. Thus the PEG additive increased the transduction efficiency by 4 and 1.6 folds for HeLa and HEK293T cells, respectively. For K562 and PC12 cells, no GFP expression was observed in the control. In the presence of 3.6% PEG additive, the transduction efficiencies of K562 and PC12 cells were increased to 25 and 71%, respectively. These results show that PEG additive can help to improve the transduction efficiency of rAAV in vitro.

We then examined the PEG concentration dependence of the transduction efficiency of rAAV virus. Cells were cultured with rAAV9-CMV::GFP solutions at an MOI of 5 × 10^5^ v·g/cell with various amounts of PEG additive. As shown in Fig. 1c-d and Supplementary Fig. 1, the GFP expression of all studied cells increased with the concentration of PEG varying from 0.2 to 3.6% (wt/vol) after a 3-day infection. The transduction efficiency dependence of rAAV on PEG molecular weight (MW) was also investigated. As shown in Supplementary Fig. 2a-b, all of the PEGs can enhance the transduction efficiency of rAAV in HeLa cells. However, the cell counting kit-8 (CCK-8) assay results indicated that the cytotoxicity of PEG increases with the increment of MW and the concentration of PEG additives (Supplementary Fig. 2c). Thus MW4000 with concentration under 3.6% is an optimal molecular weight and concentration that induces high enhancement efficiency but low cytotoxicity. Additionally, mouse brain-originated astrocyte (C8-D1A) was transfected with rAAV9-GFAP::EGFP with PEG additives at the concentrations of 0, 1.6 and 3.6% and 5.4%, respectively, and similar enhancement effect was obtained (Supplementary Fig. 3). The transduction ratio can reach to ~ 40% when the concentration of PEG is 3.6%, and reach to ~ 90% when the concentration of PEG is 5.4%. These results indicate that PEG can be used as an additive to enhance the transduction efficiency of rAAV virus, which is generally applicable for diverse cell lines and rAAV promoters.

Finally, we investigated the mechanism of PEG-enhanced transduction efficiency of rAAV vectors. We performed transmission electron microscope (TEM) to determine whether PEG and rAAV formed complexes in mixed transduction solutions. As shown in Supplementary Fig. 4, the rAAV virus from the rAAV9-CMV::GFP/PEG solutions appeared as uniform particles with a diameter of ~ 20 ± 1.5 nm and were essentially identical to untreated rAAV virus, thus indicating that PEG did not interact with rAAV virus to form complexes. More detailed studies should be conducted to elucidate the mechanism of PEG-enhanced transduction.

### PEG-improved rAAV expression in brain

**Fig. 2:**
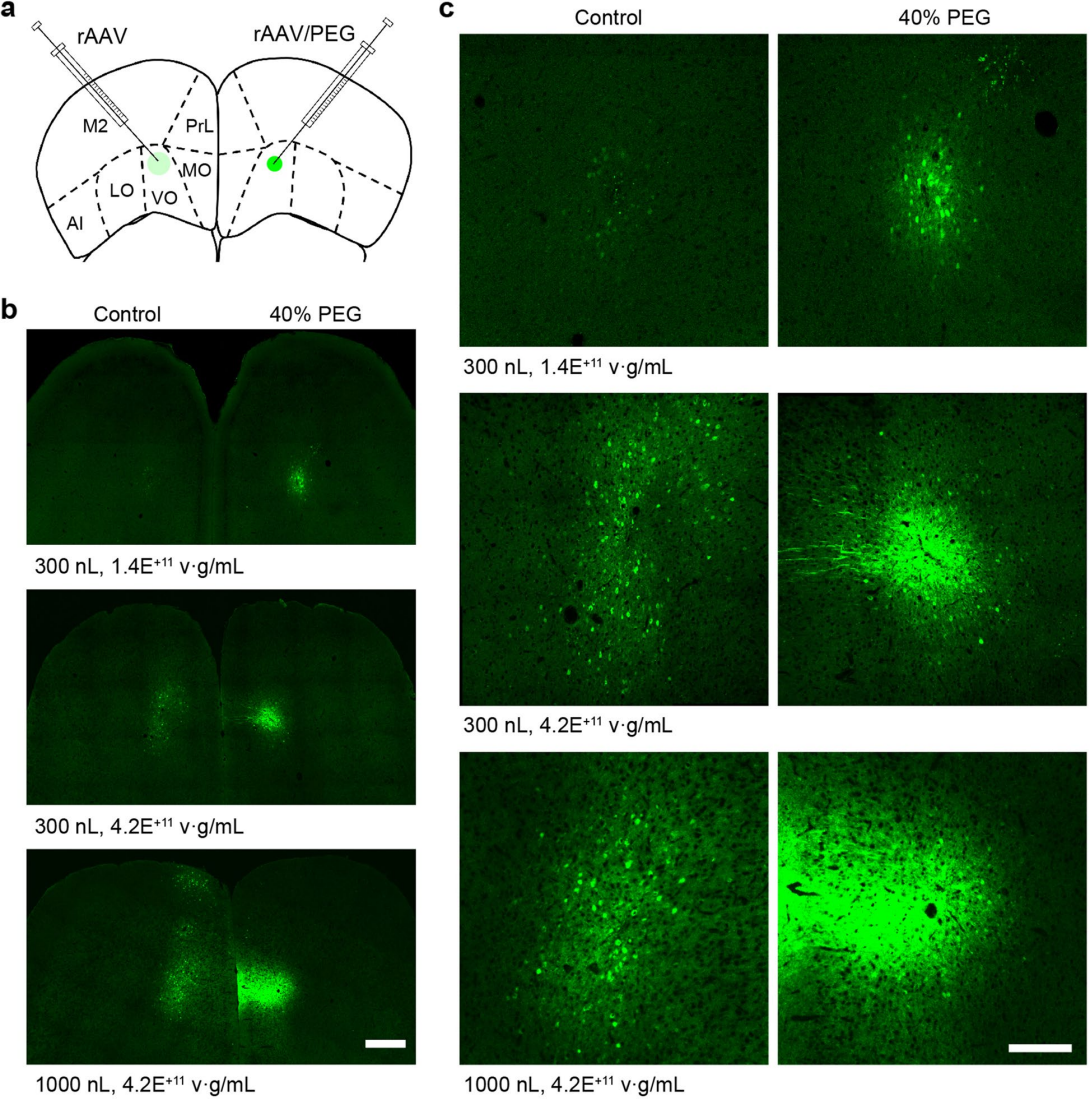
PEG-enhanced transduction of rAAV9-hSyn::EGFP in vivo. **a** A schematic showing an intraparenchymal injection of rAAV and rAAV/PEG in the same mouse brain. **b** Transgene expression of rAAV (left) and rAAV/40% (wt/vol) PEG (right). Scale bar, 500 μm. **c** Enlarged view of EGFP expression in (**b)**. Scale bar, 200 μm

The above results have demonstrated that PEG can enhance the in vitro transduction efficiency of rAAV in cultured cells. Next, we examined the effects of PEG on rAAV-mediated transduction in the mouse brain. rAAV9 vectors that carried the gene encoding enhanced green fluorescent protein (EGFP) under the control of neuron-specific human synapsin-1 promoter (rAAV9-hSyn::EGFP), were injected into the secondary motor cortex (M2) of mice. 300 nL of 1.4 × 10^11^ v·g/mL rAAV9 solutions with 40% (wt/vol) PEG were injected to the right hemispheres of the mouse brains, whereas the control rAAV9 solutions without PEG were injected into the contralateral hemispheres (Fig. 2a). After 3 weeks of gene expression, the mouse brains were sectioned for EGFP expression analysis. As shown in Fig. 2b-c, EGFP fluorescence was barely detectable in the side of untreated rAAV at a low titer of 1.4 × 10^11^ v·g/mL. On the other hand, bright EGFP fluorescence was observed in the side of 40% PEG-treated rAAV at the same titer. In addition, we found the EGFP fluorescence intensity increased when the concentrations of PEG increased from 10 to 40% (wt/vol) (Supplementary Fig. 5). Note that further increasing the PEG concentration could result in failures of rAAV injection because of the increased viscous force, especially when the diameter of syringe is small. When we increased the titer and volume of the rAAV solutions to 4.2 × 10^11^ v·g/mL and 1 μL (Fig. 2b-c and Supplementary Figs. 6–7), EGFP expression was weak and diffusive in the side injected with untreated rAAV solutions. On the other hand, strong and concentrated EGFP expression was observed in the side injected with PEG-treated rAAV solutions. These results indicate that PEG additive could enable a spatially confined and efficient expression of low-titer rAAV in the brain.

**Fig. 3:**
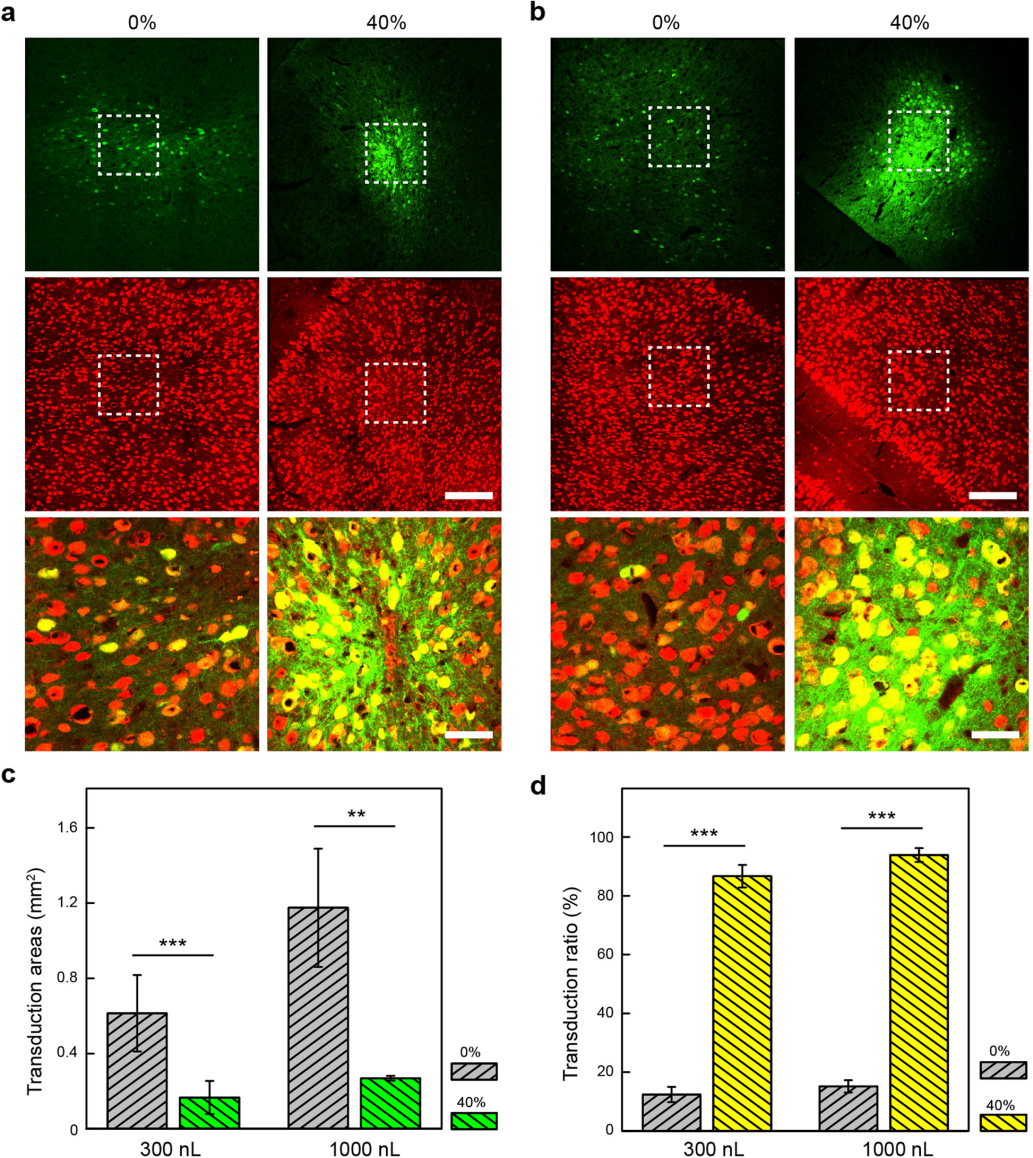
The spatially confined and high-efficient transduction of PEG-treated rAAV9-hSyn::EGFP in vivo. **a** EGFP expression and immunohistochemical staining images of brain slices with rAAV (left) and rAAV/40% (wt/vol) PEG (right) injection at the volume of 300 nL. From top to bottom: EGFP; NeuN used to label the nuclei of mature neurons; overlay images. Scale bars, 200 μm and 50 μm. **b** EGFP expression and immunohistochemical staining images of brain slices with rAAV (left) and rAAV/40% PEG (right) injection at the volume of 1000 nL. From top to bottom: EGFP; NeuN used to label the nuclei of mature neurons; overlay images. Scale bars, 200 μm and 50 μm. **c** Transduction areas of rAAV and rAAV/40% PEG. The average transduction areas and standard deviation were calculated from three or two mice and three brain slices were selected from each mouse. **P < 0.01, ***P < 0.001, unpaired two-tailed t-test. **d** Transduction efficiency of rAAV and rAAV/40% PEG. The ratio between the number of EGFP^+^NeuN^+^ cells and the total number of NeuN^+^ cells within the region of EGFP^+^ cells was calculated as transduction efficiency. The average transduction efficiency and standard deviation were calculated from two mice and three brain slices from each mouse. ***P < 0.001, unpaired two-tailed t-test

We labeled neuronal cells in the brain with the neuron-specific marker NeuN to quantitatively evaluate the effects of PEG on rAAV transduction (Fig. 3a-b). It was found that the addition of PEG could promote the expression of rAAV in a spatially confined region (Fig. 3c). At rAAV titer of 4.2 × 10^11^ v·g/mL, the transduction areas are 0.61 ± 0.20 mm^2^ and 1.18 ± 0.31 mm^2^ for 300-nL and 1000-nL injection, respectively, in the absence of PEG. With 40% PEG additive, the transduction areas are reduced to 0.17 ± 0.09 mm^2^ and 0.27 ± 0.01 mm^2^ for 300-nL and 1000-nL injection, respectively. To determine the transduction efficiency, we measured the ratio of EGFP^+^NeuN^+^ cells to NeuN^+^ cells within the expression areas of EGFP (Fig. 3d). At rAAV titer of 4.2 × 10^11^ v·g/mL, the transduction ratio of 300-nL intraparenchymal administration was (12.4 ± 2.6) % in the absence of PEG, compared to (86.6 ± 3.8) % in the presence of 40% PEG. With 1000-nL intraparenchymal administration, the transduction efficiency of rAAV was (15.2 ± 2.1) % in the absence of PEG and (93.9 ± 2.3) % in the presence of 40% PEG. These results confirm that the PEG additive can result in highly efficient and localized transduction of rAAV vectors in the brain.

### The biocompatibility of PEG in brain

**Fig. 4:**
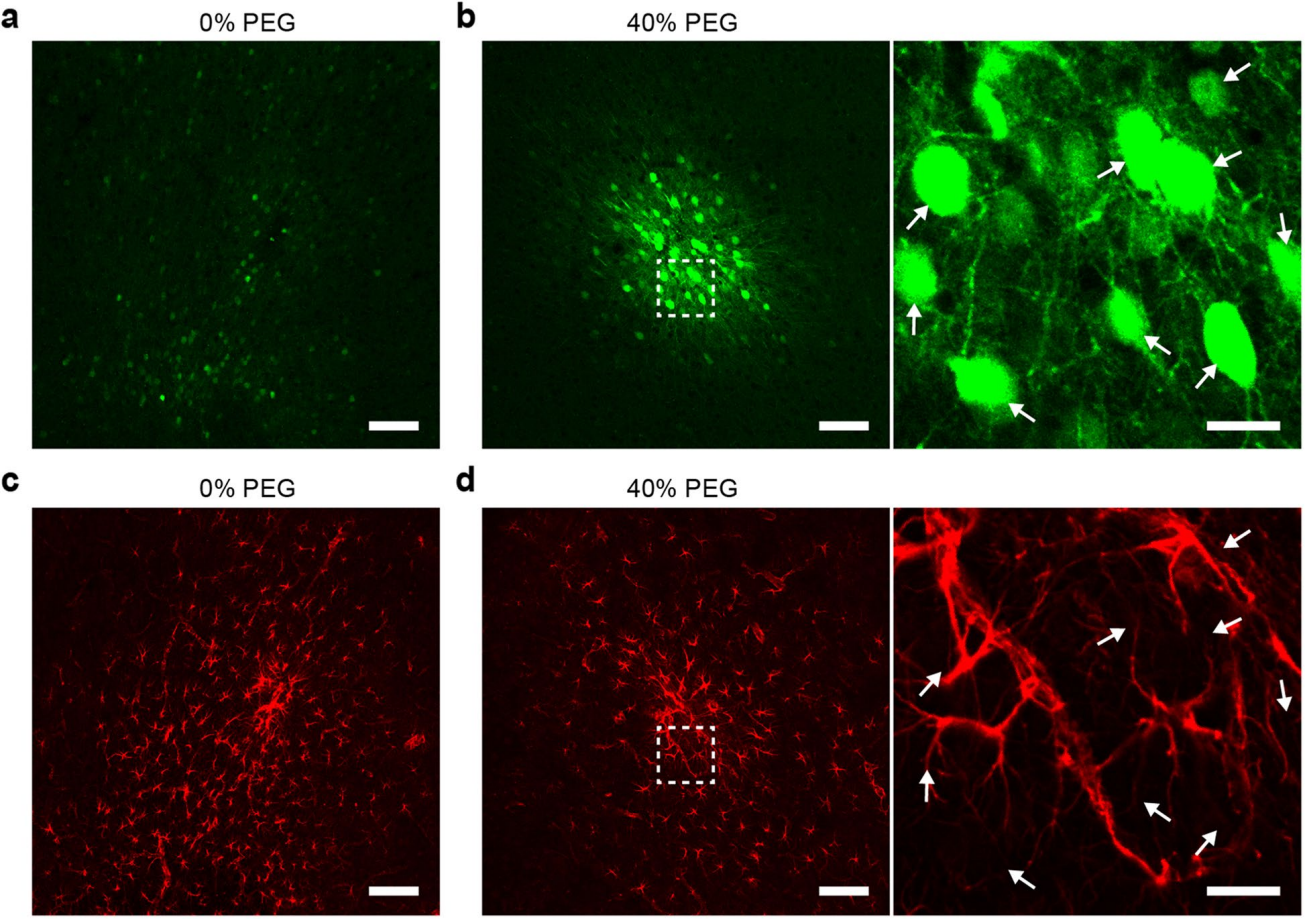
Long-term transgene expression of PEG-treated rAAV9-hSyn::EGFP in vivo. **a** EGFP expression in brain slices after an 8-week transduction of untreated rAAV9-hSyn::EGFP. Scale bar, 100 μm. **b** EGFP expression in brain slices after an 8-week transduction of 40% PEG-treated rAAV9-hSyn::EGFP. The right figure shows the zoom-in view of EGFP in the white dashed box in left figure. The white arrows indicate the positions of EGFP-positive neurons. Scale bar, 100 μm and 20 μm. **c** GFAP in brain slices after an 8-week transduction of untreated rAAV. Scale bars, 100 μm. **d** GFAP in brain slices after an 8-week transduction of 40% PEG-treated rAAV. The right figure shows the zoom-in views of GFAP in the white dashed box in left figure. The white arrows indicate the positions of EGFP-positive neurons. Scale bars, 100 μm and 20 μm

Next, we examined time dependent characteristics of PEG-assisted rAAV transduction in the brain. EGFP fluorescence was characterized after 3- or 8-week expressions of untreated and 40% PEG-treated rAAV9-hSyn::EGFP at a titer of 4.2 × 10^11^ v·g/mL, respectively. As shown in Fig. 4a, EGFP expression of untreated rAAV solutions at 8 weeks after intraparenchymal injection was similar to that at 3 weeks, both of which were of poor intensity and diffusive areas. On the other hand, the EGFP expression mediated by 40% PEG-treated rAAV appeared to be in strong intensity and spatially confined areas at both 3 and 8 weeks after intraparenchymal injection (Fig. 4b). More detailed comparisons of EGFP expression in the depth direction were shown in Supplementary Fig. 8. The above results confirm that PEG can enable a spatially confined and efficient transgene expression in the brain over long terms.

In previous studies, a series of compounds have been used as rAAV transduction enhancer, including topoisomerase and proteasome inhibitors, epipodophyllotoxins, inducers of DNA damage, effectors of epigenetic modification, and anthracyclines [26, 40, 41]. However, several side effects have hindered their applications, calling for alternative agents that are more biocompatible. Next, we characterized the effects of PEG enhancer on the immune responses, cell morphology, cell tropism of rAAV, neuronal apoptosis, and motor function of animals. 300-nL rAAV9-hSyn::EGFP solutions at the titer of 4.2 × 10^11^ v·g/mL were injected into the M2 of mice with or without 40% PEG. After 8 weeks of expression, the brains were sectioned, and astrocytes were labeled by staining glial fibrillary acid protein (GFAP) to analyze the immune responses. As shown in Fig. 4c-d, no apparent difference in the density of GFAP was found between rAAV- and rAAV/PEG-transfected brain slices. We also found that neurons and astrocytes around the injection sites of rAAV/PEG solution maintained typical cell morphology. In addition, no overlay was found between EGFP^+^ cells and astrocytes, as indicated by the white arrows. These results show that no EGFP expression was leaked into astrocytes under the hSyn promoter control, confirming the good tropism of rAAV on cell types.

**Fig. 5:**
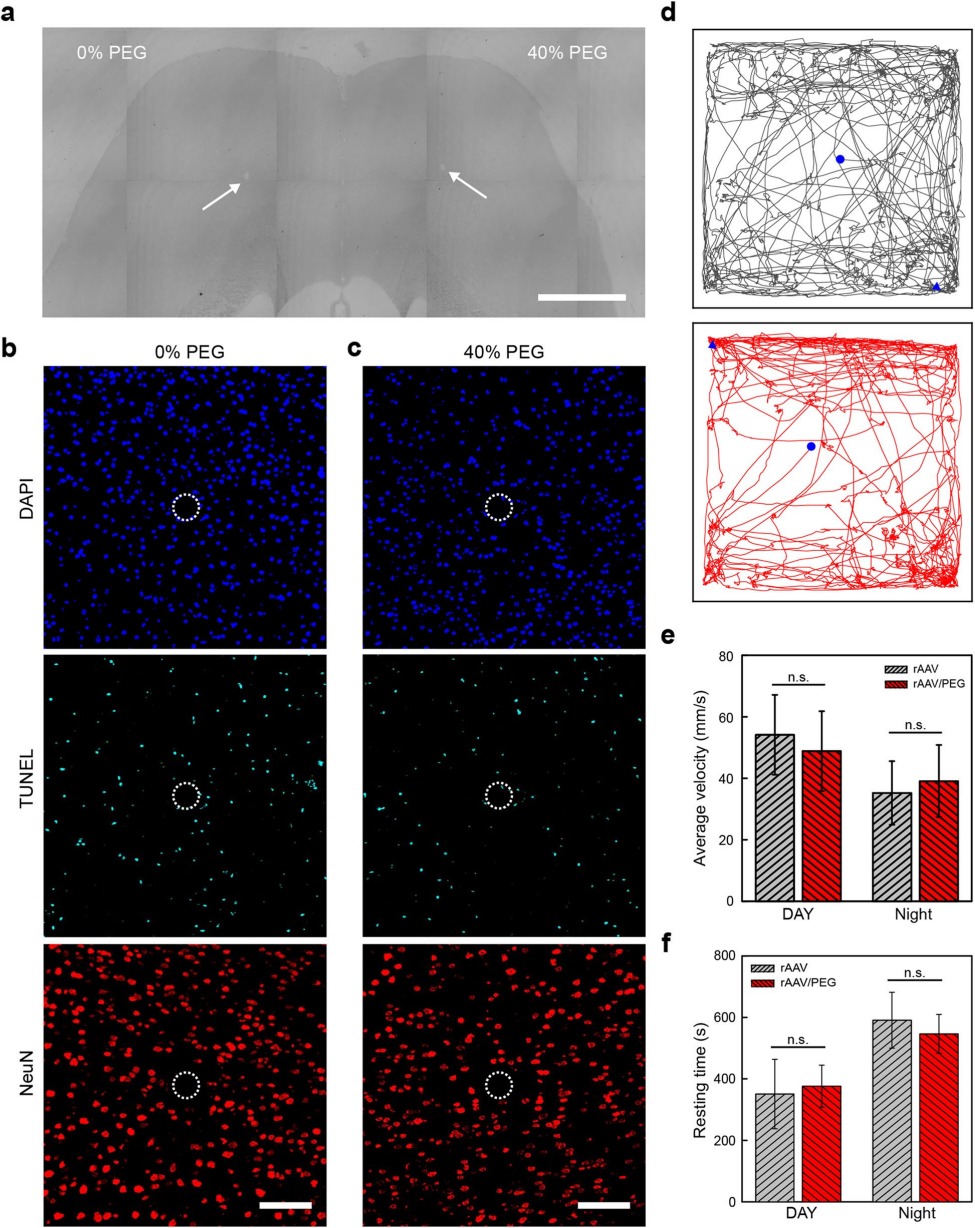
Biocompatibility assessment of PEG in the brain. **a** Bright field of a brain slice with white arrows indicating the injecting sites. The PEG concentration of injecting solution in the left and right brain is 0 and 40% (wt/vol), respectively. Scale bar, 1 mm. **b-c** Immunohistochemical staining of the brain slice in **a**. The brain slice was prepared at 3 weeks after injections and labeled for nuclei [4^′^, 6-diamidino-2-phenylindole (DAPI), blue], apoptotic cells [terminal deoxynucleotidyl transferase dUTP nick end labeling (TUNEL), cyan], and neurons (NeuN, red). The dashed circles highlight the injection sites. Scale bars, 100 μm. **d** Representative movement traces of mice with injections of untreated rAAV solutions (gray) or 40% PEG-treated rAAV solutions (red) in the M2 cortex. The start and end positions are marked by blue circles and triangles, respectively. **e-f** Average locomotion speeds **e** and resting time **f** of the two groups of mice in (**d)**. *n* = 4 mice in each group. Data are represented as mean ± SD. n.s. *P* > 0.5, unpaired two-tailed t-test

To examine the activities of PEG on neuronal apoptosis, the nuclei of all cells, apoptotic neurons, and mature neurons were labeled by staining DAPI, TUNEL and NeuN, respectively (Fig. 5a). The immunochemical results showed that the densities of cells, apoptotic neurons, and mature neurons were similar between control side and the rAAV/PEG-injected side (Fig. 5b, c). The above results confirm the good biocompatibility of PEG in the brain. We further investigated the impact of PEG on animal behaviors. In the control group, 300-nL rAAV9-hSyn::EGFP without PEG treatment was injected into the left and right M2 of mice at a titer of 4.2 × 10^11^ v·g/mL. In the experiment group, 300-nL rAAV9-hSyn::EGFP treated with 40% PEG was injected at the same titer. After a 3-week expression, the motor functions of the two groups of mice were assessed by performing 15-minute open field tests. In order to eliminate the impact of rhythm, the movements of mice were recorded by automatic video-tracking software during the day and night, respectively. As shown in Fig. 5d, the representative movement traces for the two groups of mice were random and similar. The average locomotion speeds and resting time for the two groups of mice in the 15-minute record were summarized in Fig. 5e-f, respectively. Unpaired t-tests were carried out to determine whether significant differences existed between the two groups of mice. The *P* values of the average locomotion speeds between the two groups were 0.635 and 0.685 at day and night, respectively. The *P* values of resting time between the two groups were 0.750 and 0.519 at day and night, respectively. These results demonstrated that PEG did not result in alterations of motor function in mice. In conclusion, the PEG additive at a dose that can significantly improve rAAV transduction shows good biocompatibility in the CNS.

## Discussion

The transduction efficiency of rAAV is largely depended on the cellular internalization and the following downstream events after the internalization of rAAV [42, 43]. Specifically, rAAV internalization is mainly determined by the molecular interactions between rAAV capsid and cell membrane receptor. It is well known that PEG is a type of super hydrophilic polymer that can bind water with extremely high affinity, so it was speculated that the PEG produced a pronounced volume exclusion effect to reduce the distance between rAAV vectors and cells. In addition, the PEG molecule is known to dramatically enhance the fluidity of the cell membrane, and has been applied as an accelerator for cellular fusion in previous studies [44]. These capabilities may facilitate rAAV particles entering protoplasm, thus promoting the uptake of rAAV in targeted cells during the diffusion of rAAV solutions. In this case, the endocytosis of rAAV is accelerated and the spreading distance of rAAV vectors is reduced, leading to the locally concentrated transgene expression.

After internalization, two main steps still need to go through before the transgene expression occurs in the host cells, including the release of virion core into the cytoplasm, and the transcription of the viral single-stranded RNA to double-stranded DNA. It was reported that the PEG can induce the condensation and packaging of DNA in the presence of Na^+^ or Mg^2+^ salts [45, 46], and this polymer-assisted compaction can protect DNA from protein-DNA interactions, such as enzymatic degradation, immunostimulation, et al. [47]. Taken together, it is possible that the PEG increased the amount of rAAV getting into the cell, as well as the proportion of rAAV genomes coming to transcription, both of which reinforced the expression of viral DNA in targeted cells.

Finally, the enhanced transduction efficiency of rAAV could benefit partially from physical processes. For the in vivo transduction, the addictive of PEG could increase the viscosity of rAAV solutions, thus decreasing the diffusion behavior of rAAV in tissue.

## Conclusions

In this work, we developed an easy and effective method by using PEG as auxiliary agent to improve the rAAV transduction characteristics both in vitro and in vivo. Our results showed that the in vitro expression of rAAV was significantly enhanced by adding a small dose of PEG. Especially, an efficient transgene expression of rAAV in was achieved in the presence of PEG for cell lines that are difficult to be transfected with rAAV alone. For in vivo transduction experiments, rAAV9-hSyn::EGFP solutions with or without PEG were injected into mouse brains at low titers. After both 3 and 8 weeks of expression, the PEG additive promoted a spatially confined and efficient transduction in the mouse brains. In addition, the PEG additive did not cause any detectable side effects in the brain, including aggravated immune responses, abnormal cell morphology, alteration of rAAV tropism, cytotoxicity, and animal behavior alteration. To summary, we demonstrated a simple strategy of using PEG as transduction auxiliary to improve the expression of rAAV in vitro and in vivo, especially in the CNS. We also demonstrated that the improvement of rAAV transduction in vivo could persist for at least 2 months without measurable side effects. Our strategy broadens the applications of rAAV in both scientific research and clinical therapy, especially when it is necessary to transfer genes to small nuclei of brain with high precision and efficiency.

## Supplementary Information

**Additional file 1: Supplementary Fig. 1.** PEG-enhanced infection of rAAV-CMV::GFP vectors in HEK293T, K562 and PC12 cells. (a) Transgene expression of rAAV9-CMV::GFP in HEK293T (left), K562 (middle) and PC12 (right) cells with different concentrations of PEG excipient. The MOI was kept at 5 × 10^5^ v·g/cell, and the concentrations of PEG excipient were 0.2, 0.8, 1.6, and 3.6% (wt/vol) from top to bottom, respectively. Columns from left to right: green fluorescence, bright field, and overlay images. Scale bars, 100 μm. (b) Dependence of the transduction efficiency of rAAV9-CMV::GFP in HEK293T (left), K562 (middle) and PC12 (right) cells on the concentration of PEG excipient. The MOI was kept at 5 × 10^5^ v·g/cell. *n* = 3 wells in each group. Data are represented as mean ± SD. **Supplementary Fig. 2.** Molecular weight dependence of infection ability of PEG/rAAV vectors in vitro. (a) GFP expression in HeLa cell line transduced with untreated rAAV9-CMV::GFP (top) and rAAV9-CMV::GFP with PEG2000, PEG4000, and PEG10000 (bottom), respectively, at the MOI of 5 × 10^5^ v·g/cell. Scale bars, 200 μm. (b) Dependence of the transduction efficiency of rAAV9-CMV::GFP in HeLa cells on the concentration of different PEG additives. *n* = 3 wells in each group. Data are represented as mean ± SD. (c) CCK-8 assay of HeLa cell line that incubated with culture medium with different concentration of PEG2000, PEG4000, and PEG10000 additives, respectively. **Supplementary Fig. 3.** PEG-enhanced infection of rAAV-GFAP::EGFP vectors in C8-D1A cell line in vitro. (a) EGFP expression in C8-D1A cell line transduced with untreated rAAV9-GFAP::EGFP and rAAV9-GFAP::EGFP treated with 1.6, 3.6 and 5.4% (wt/vol) PEG additive, respectively, at the MOI of 2 × 10^6^ v·g/cell. Scale bar, 200 μm. (b) Abnormal cell morphology occurred in C8-D1A when the concentration of PEG ratio was 7.2%. Scale bar, 200 μm. (c) Dependence of the transduction efficiency of rAAV9-GFAP::EGFP in C8-D1A cell on the concentration of PEG additive. The MOI was kept at 2 × 10^6^ v·g/cell. *n* = 3 wells in each group. Data are represented as mean ± SD. **Supplementary Fig. 4.** TEM characterizations of rAAV9-CMV::GFP virus. Negative stain of rAAV9-CMV::GFP virus from rAAV9-CMV::GFP (left) and rAAV9-CMV::GFP/PEG solutions (right), respectively. TEM samples of rAAV vectors were prepared by loading the copper grids (250-mesh, coated with a formvar-thin carbon film) with 10 μL pure rAAV solution or rAAV/PEG solution at a titer of 10^11^ v·g/mL, and then stained with 5% uranyl acetate. The concentrations of PEG4000 in the rAAV/PEG solution were 20%. Scale bar, 200 nm. **Supplementary Fig. 5.** 3-week expression of 300-nL rAAV9-hSyn::EGFP in mice at a titer of 1.4 × 10^11^ v·g/mL. The concentrations of PEG from left to right are 0, 10, 20, 30, and 40%, respectively. Scale bar, 100 μm. **Supplementary Fig. 6.** 3-week expression of 300-nL rAAV9-hSyn::EGFP in mice at a titer of 4.2 × 10^11^ v·g/mL. The concentrations of PEG from left to right are 0, 20, and 40%, respectively. Scale bar, 200 μm. **Supplementary Fig. 7.** 3-week expression of 1000-nL rAAV9-hSyn::EGFP in mice at a titer of 4.2 × 10^11^ v·g/mL. The concentrations of PEG in the left and right are 0 and 40%, respectively. Scale bar, 200 μm. **Supplementary Fig. 8.** Transduction comparison of rAAV9-hSyn::EGFP at 3 weeks (a) and 8 weeks (b) after intraparenchymal injection. For precise comparison, the same tube of rAAV or rAAV/PEG solutions were injected to all the mouse in 3-week group and 8-week group on the same day. The injection volume is 300 nL and the titer of rAAV vectors is 4.2 × 10^11^ v·g/mL. The concentrations of PEG in the left and right are 0 and 40%, respectively. Scale bars, 200 μm.

## Data Availability

The datasets used and/or analyzed during the current study are available from the corresponding author on reasonable request.

## Abbreviations

CNS: Central nervous system
AAV: Adeno-associated virus
scAAV: Self-complementary adeno-associated virus (scAAV)
PEG: Polyethylene glycol
