# Supplementary material for: PEG-mediated transduction of rAAV as a platform for spatially confined and efficient gene delivery
Source: Biomater Res. 2022 Dec 2;26:69. doi: 10.1186/s40824-022-00322-1 (PMC9716683; doi:10.1186/s40824-022-00322-1)
Supplement: Supplementary file 1 — Additional file 1: Supplementary Fig. 1. PEG-enhanced infection of rAAV-CMV::GFP vectors in HEK293T, K562 and PC12 cells. (a) Transgene expression of rAAV9-CMV::GFP in HEK293T (left), K562 (middle) and PC12 (right) cells with different concentrations of PEG excipient. The MOI was kept at 5 × 10^5^ v·g/cell, and the concentrations of PEG excipient were 0.2, 0.8, 1.6, and 3.6% (wt/vol) from top to bottom, respectively. Columns from left to right: green fluorescence, bright field, and overlay images. Scale bars, 100 μm. (b) Dependence of the transduction efficiency of rAAV9-CMV::GFP in HEK293T (left), K562 (middle) and PC12 (right) cells on the concentration of PEG excipient. The MOI was kept at 5 × 10^5^ v·g/cell. n = 3 wells in each group. Data are represented as mean ± SD. Supplementary Fig. 2. Molecular weight dependence of infection ability of PEG/rAAV vectors in vitro. (a) GFP expression in HeLa cell line transduced with untreated rAAV9-CMV::GFP (top) and rAAV9-CMV::GFP with PEG2000, PEG4000, and PEG10000 (bottom), respectively, at the MOI of 5 × 10^5^ v·g/cell. Scale bars, 200 μm. (b) Dependence of the transduction efficiency of rAAV9-CMV::GFP in HeLa cells on the concentration of different PEG additives. n = 3 wells in each group. Data are represented as mean ± SD. (c) CCK-8 assay of HeLa cell line that incubated with culture medium with different concentration of PEG2000, PEG4000, and PEG10000 additives, respectively. Supplementary Fig. 3. PEG-enhanced infection of rAAV-GFAP::EGFP vectors in C8-D1A cell line in vitro. (a) EGFP expression in C8-D1A cell line transduced with untreated rAAV9-GFAP::EGFP and rAAV9-GFAP::EGFP treated with 1.6, 3.6 and 5.4% (wt/vol) PEG additive, respectively, at the MOI of 2 × 10^6^ v·g/cell. Scale bar, 200 μm. (b) Abnormal cell morphology occurred in C8-D1A when the concentration of PEG ratio was 7.2%. Scale bar, 200 μm. (c) Dependence of the transduction efficiency of rAAV9-GFAP::EGFP in C8-D1A cell on the concentration of PEG additive. T [file 40824_2022_322_MOESM1_ESM.docx]

**SUPPLEMENTARY INFORMATION for**

**PEG-mediated transduction of rAAV as a platform for spatially confined and efficient gene delivery**

Liang Zou^1,2,3^, Jinfen Wang^1^, Ying Fang^1,2,3^, Huihui Tian^1^*

^1^CAS Center for Excellence in Nanoscience, National Center for Nanoscience and Technology, Beijing 100190, China

^2^CAS Center for Excellence in Brain Science and Intelligence Technology, Institute of Neuroscience, Chinese Academy of Sciences, Shanghai 200031, China

^3^University of Chinese Academy of Sciences, Beijing 100049, China

E-mail: tianhh@nanoctr.cn

**The PDF file includes:**

**Supplementary Figure 1.** PEG-enhanced infection of rAAV-CMV::GFP vectors in HEK293T, K562 and PC12 cells.

**Supplementary Figure 2.** Molecular weight dependence of infection ability of PEG/rAAV vectors in vitro.

**Supplementary Figure 3.** PEG-enhanced infection of rAAV-GFAP::EGFP vectors in C8-D1A cell line *in vitro*.

**Supplementary Figure 4.** TEM characterizations of rAAV9-CMV::GFP virus.

**Supplementary Figure 5.** 3-week expression of 300-nL rAAV9-hSyn::EGFP in mice at a titer of 1.4 × 10^11^ v·g/mL.

**Supplementary Figure 6.** 3-week expression of 300-nL rAAV9-hSyn::EGFP in mice at a titer of 4.2 × 10^11^ v·g/mL.

**Supplementary Figure 7.** 3-week expression of 1000-nL rAAV9-hSyn::EGFP in mice at a titer of 4.2 × 10^11^ v·g/mL.

**Supplementary Figure 8.** Transduction comparison of rAAV9-hSyn::EGFP at 3-week (a) and 8-week (b) post intraparenchymal injection.

**
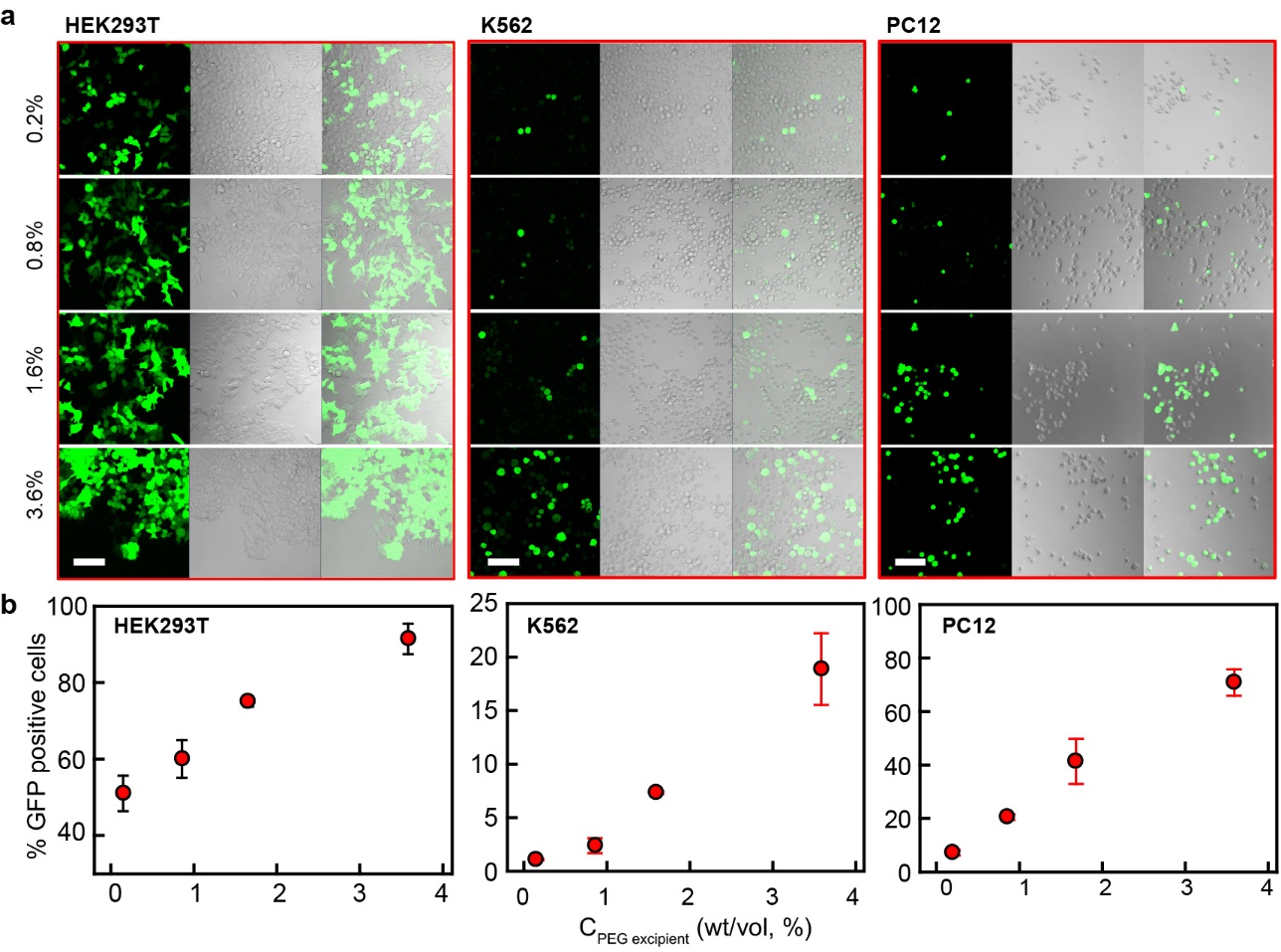
**

**Supplementary Figure 1. PEG-enhanced infection of rAAV-CMV::GFP vectors in** **HEK293T, K562 and PC12 cells.** (**a**) Transgene expression of rAAV9-CMV::GFP in HEK293T (left), K562 (middle) and PC12 (right) cells with different concentrations of PEG excipient. The MOI was kept at 5×10^5^ v·g/cell, and the concentrations of PEG excipient were 0.2%, 0.8%, 1.6%, and 3.6% (wt/vol) from top to bottom, respectively. Columns from left to right: green fluorescence, bright field, and overlay images. Scale bars, 100 μm. (**b**) Dependence of the transduction efficiency of rAAV9-CMV::GFP in HEK293T (left), K562 (middle) and PC12 (right) cells on the concentration of PEG excipient. The MOI was kept at 5 × 10^5^ v·g/cell. n = 3 wells in each group. Data are represented as mean ± SD.


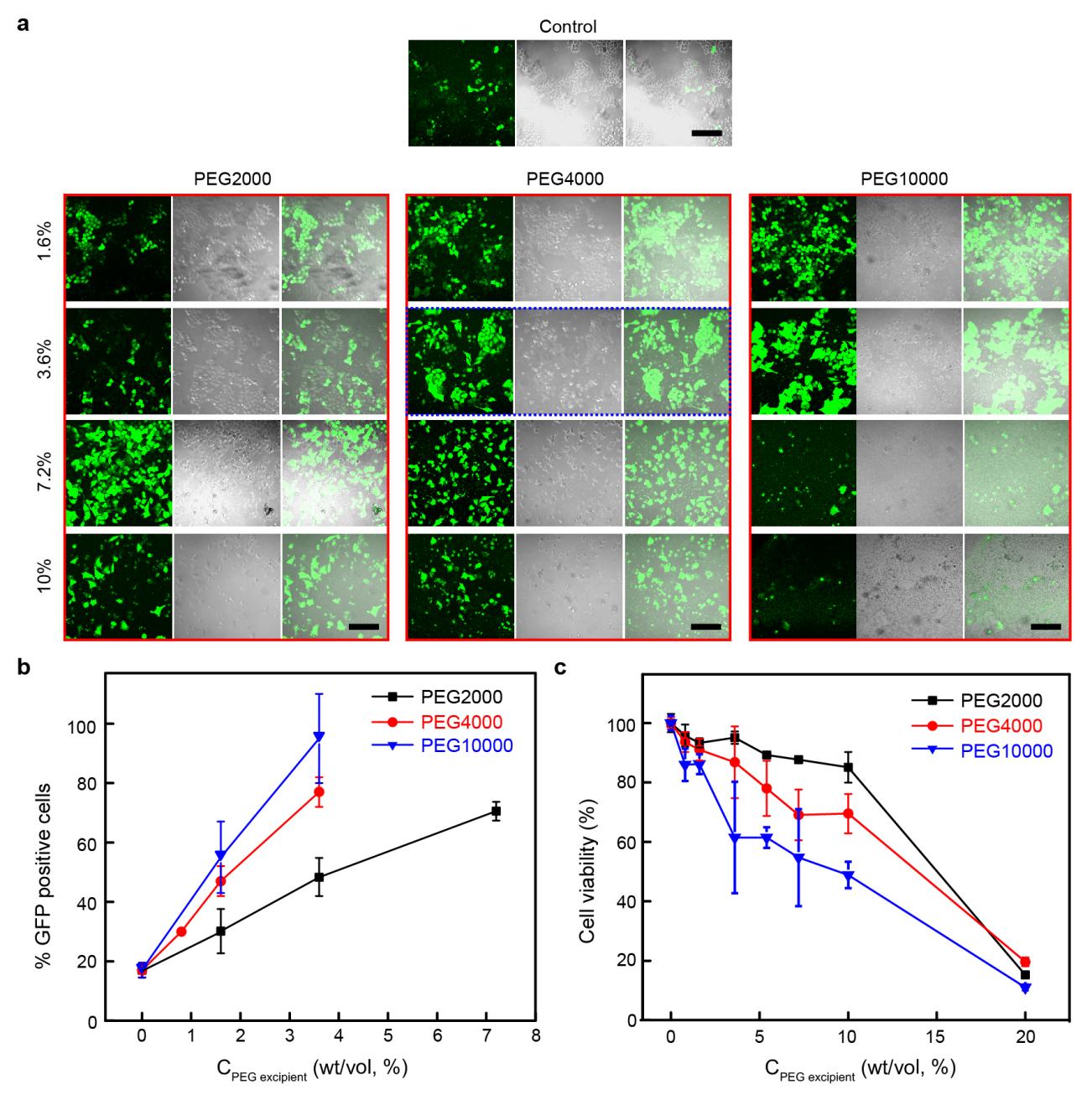


**Supplementary Figure 2. Molecular weight dependence of infection ability of PEG/rAAV vectors in vitro.** (**a**) GFP expression in HeLa cell line transduced with untreated rAAV9-CMV::GFP (top) and rAAV9-CMV::GFP with PEG2000, PEG4000, and PEG10000 (bottom), respectively, at the MOI of 5 × 10^5^ v·g/cell. Scale bars, 200 µm. **(b)** Dependence of the transduction efficiency of rAAV9-CMV::GFP in HeLa cells on the concentration of different PEG additives. n = 3 wells in each group. Data are represented as mean ± SD. **(c)** CCK-8 assay of HeLa cell line that incubated with culture medium with different concentration of PEG2000, PEG4000, and PEG10000 additives, respectively.


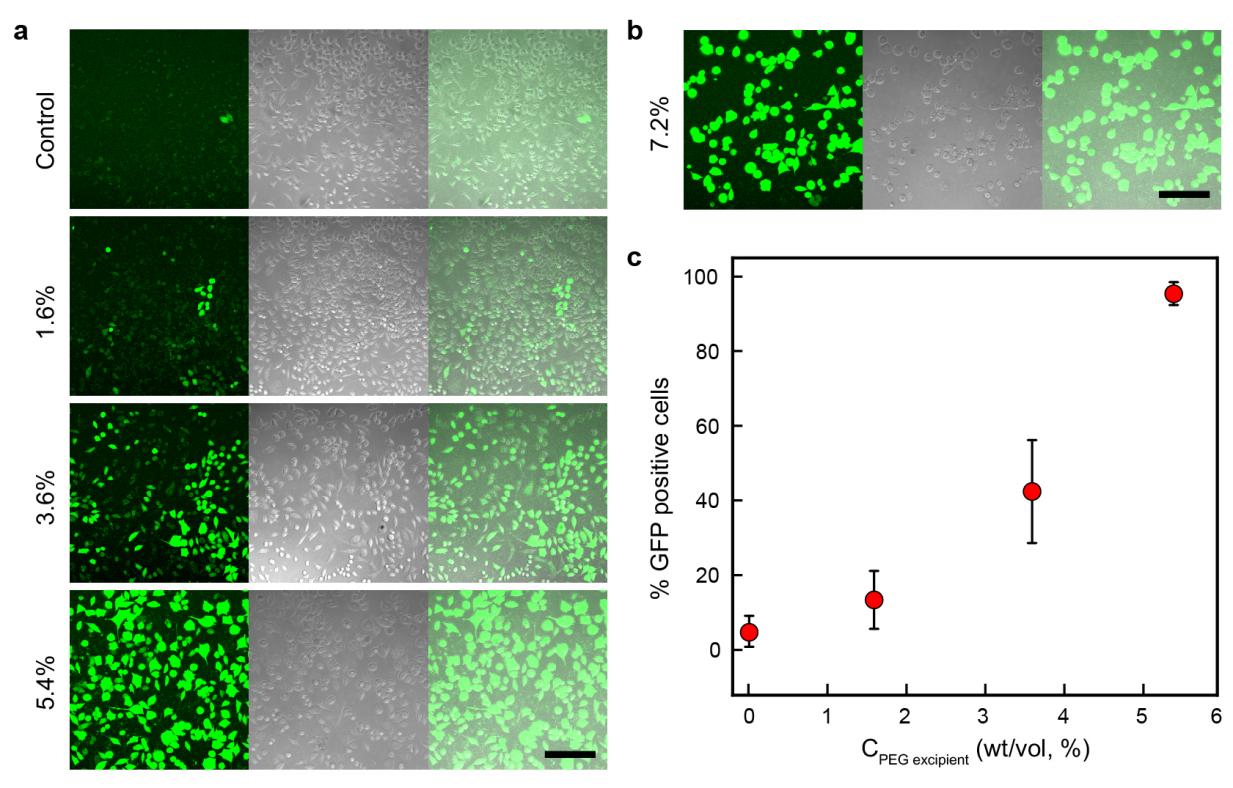


**Supplementary Figure 3. PEG-enhanced infection of rAAV-GFAP::EGFP vectors in C8-D1A cell line *in vitro*. (a)** EGFP expression in C8-D1A cell line transduced with untreated rAAV9-GFAP::EGFP and rAAV9-GFAP::EGFP treated with 1.6%, 3.6% and 5.4% (wt/vol) PEG additive, respectively, at the MOI of 2 × 10^6^ v·g/cell. Scale bar, 200 μm. (b) Abnormal cell morphology occurred in C8-D1A when the concentration of PEG ratio was 7.2%. Scale bar, 200 μm. (c) Dependence of the transduction efficiency of rAAV9-GFAP::EGFP in C8-D1A cell on the concentration of PEG additive. The MOI was kept at 2 × 10^6^ v·g/cell. n = 3 wells in each group. Data are represented as mean ± SD.

**
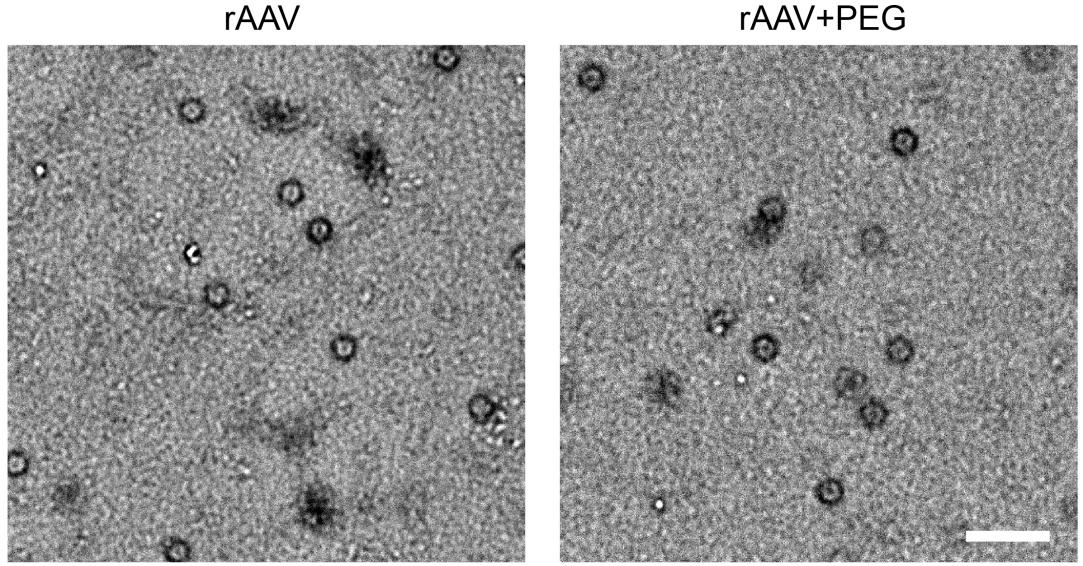
**

**Supplementary Figure 4. TEM characterizations of rAAV9-CMV::GFP virus.** Negative stain of rAAV9-CMV::GFP virus from rAAV9-CMV::GFP (left) and rAAV9-CMV::GFP/PEG solutions (right), respectively. TEM samples of rAAV vectors were prepared by loading the copper grids (250-mesh, coated with a formvar-thin carbon film) with 10 μL pure rAAV solution or rAAV/PEG solution at a titer of 10^11^ v·g/mL, and then stained with 5% uranyl acetate. The concentrations of PEG 4000 in the rAAV/PEG solution were 20%. Scale bar, 200 nm.

**
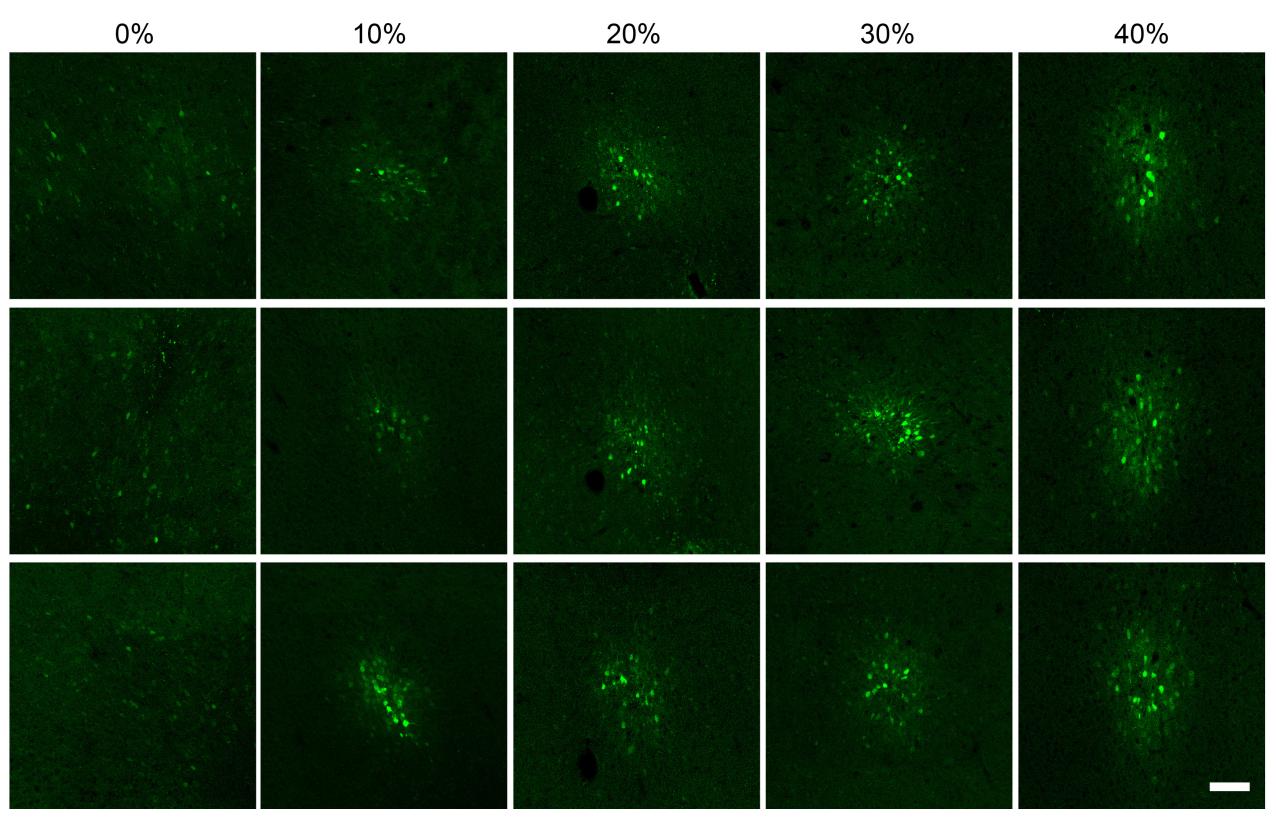
**

**Supplementary Figure 5. 3-week expression of 300-nL rAAV9-hSyn::EGFP in mice at a titer of 1.4 × 10^11^ v·g/mL.** The concentrations of PEG from left to right are 0%, 10%, 20%, 30%, and 40%, respectively. Scale bar, 100 μm.

**
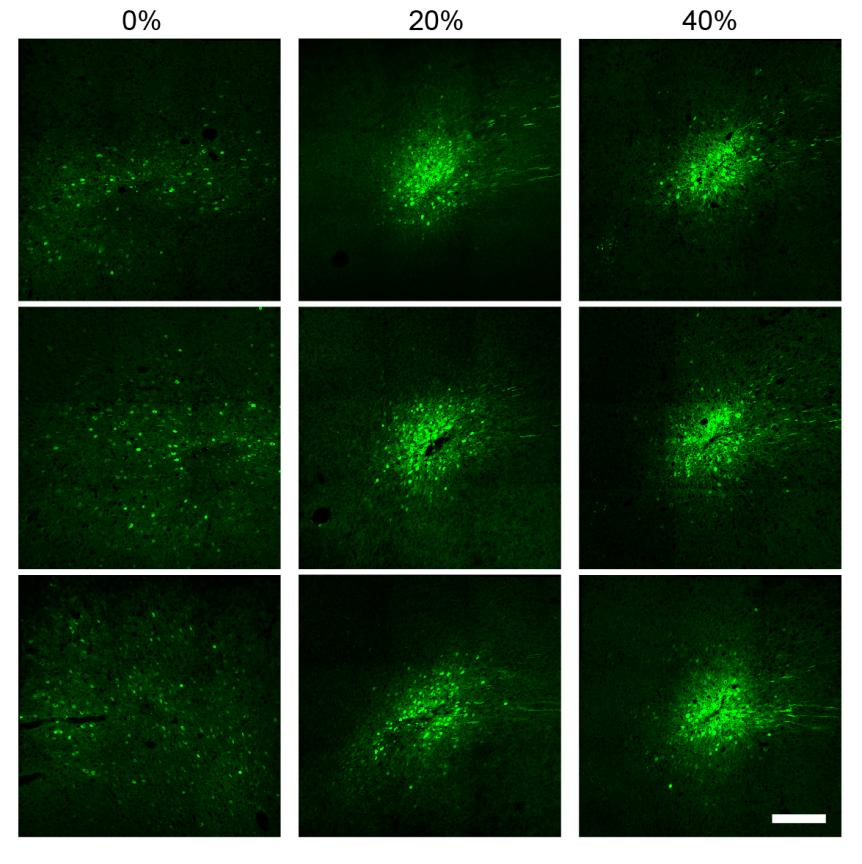
**

**Supplementary Figure 6. 3-week expression of 300-nL rAAV9-hSyn::EGFP in mice at a titer of 4.2** × **10^11^ v·g/mL.** The concentrations of PEG from left to right are 0%, 20%, and 40%, respectively. Scale bar, 200 μm.

**
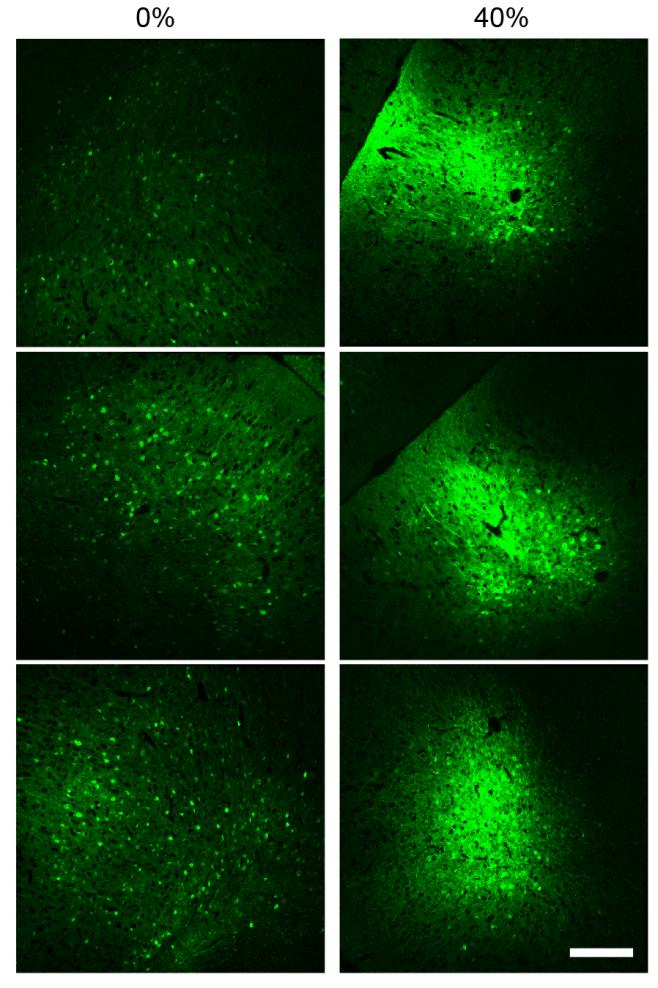
**

**Supplementary Figure 7. 3-week expression of 1000-nL rAAV9-hSyn::EGFP in mice at a titer of 4.2** × **10^11^ v·g/mL.** The concentrations of PEG in the left and right are 0% and 40%, respectively. Scale bar, 200 μm.

**
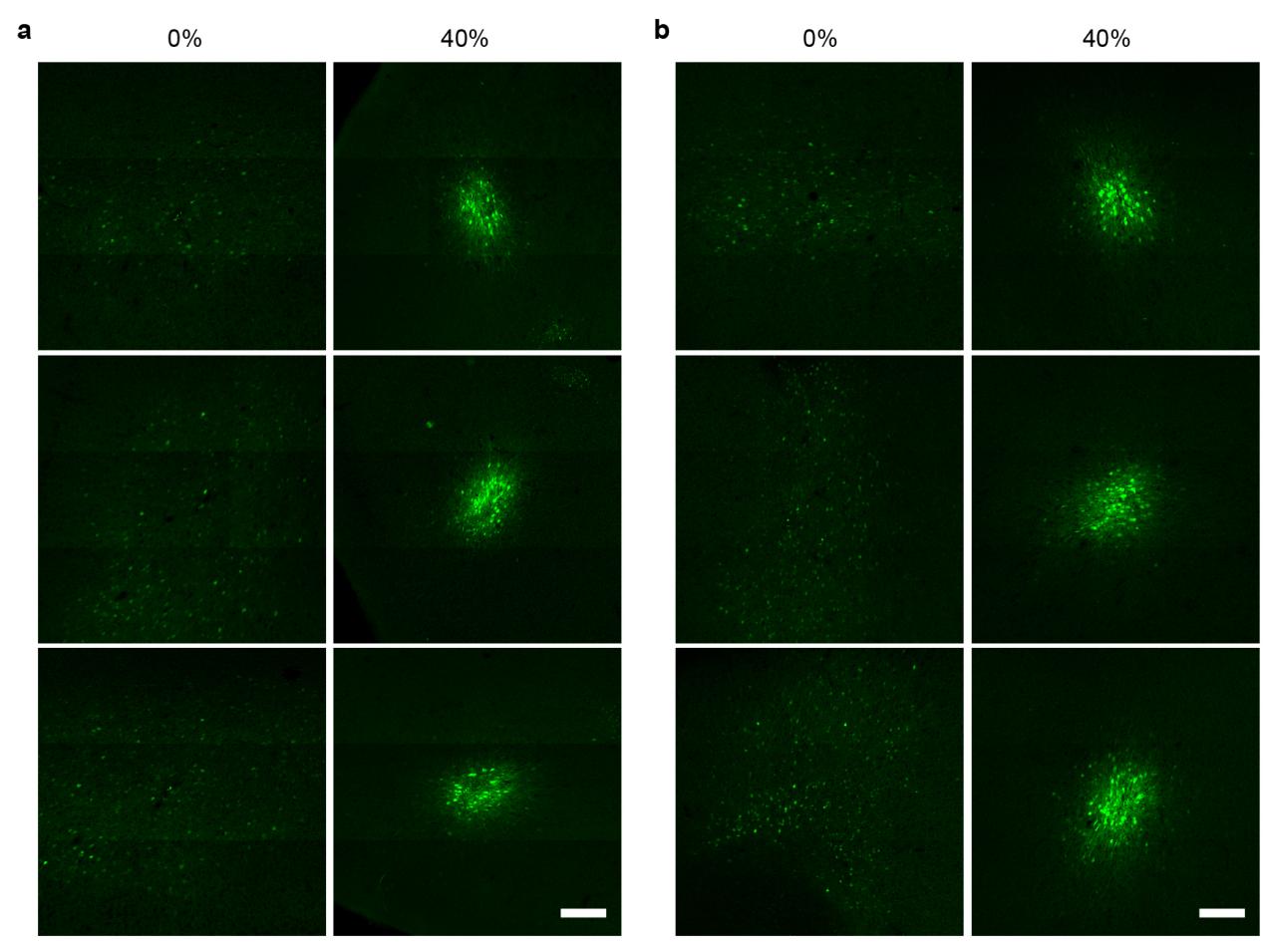
**

**Supplementary Figure 8. Transduction comparison of rAAV9-hSyn::EGFP at 3-week (a) and 8-week (b) post intraparenchymal injection.** For precise comparison, the same tube of rAAV or rAAV/PEG solutions were injected to all the mouse in 3-week group and 8-week group on the same day. The injection volume is 300 nL and the titer of rAAV vectors is 4.2 × 10^11^ v·g/mL. The concentrations of PEG in the left and right are 0% and 40%, respectively. Scale bars, 200 μm.
